# Bidirectional Ductal Shunting and Preductal to Postductal Oxygenation Gradient in Persistent Pulmonary Hypertension of the Newborn

**DOI:** 10.3390/children7090137

**Published:** 2020-09-15

**Authors:** Amy Lesneski, Morgan Hardie, William Ferrier, Satyan Lakshminrusimha, Payam Vali

**Affiliations:** Davis School of Medicine, University of California, Sacramento, CA 95817, USA; allesneski@ucdavis.edu (A.L.); mehardie@ucdavis.edu (M.H.); wtferrier@ucdavis.edu (W.F.); slakshmi@ucdavis.edu (S.L.)

**Keywords:** oxygenation saturation, patent ductus arteriosus, pulmonary hypertension

## Abstract

Background: The aim was to evaluate the relationship between the direction of the patent ductus arteriosus (PDA) shunt and the pre- and postductal gradient for arterial blood gas (ABG) parameters in a lamb model of meconium aspiration syndrome (MAS) with persistent pulmonary hypertension of the newborn (PPHN). Methods: PPHN was induced by intermittent umbilical cord occlusion and the aspiration of meconium through the tracheal tube. After delivery, 13 lambs were ventilated and simultaneous 129 pairs of pre- and postductal ABG were drawn (right carotid and umbilical artery, respectively) while recording the PDA and the carotid and pulmonary blood flow. Results: Meconium aspiration resulted in hypoxemia. The bidirectional ductal shunt had a lower postductal partial arterial oxygen tension ([PaO_2_] with lower PaO_2_/FiO_2_ ratio—97 ± 36 vs. 130 ± 65 mmHg) and left pulmonary flow (81 ± 52 vs. 133 ± 82 mL/kg/min). However, 56% of the samples with a bidirectional shunt had a pre- and postductal saturation gradient of < 3%. Conclusions: The presence of a bidirectional ductal shunt is associated with hypoxemia and low pulmonary blood flow. The absence of a pre- and postductal saturation difference is frequently observed with bidirectional right-to-left shunting through the PDA, and does not exclude a diagnosis of PPHN in this model.

## 1. Introduction

Persistent pulmonary hypertension of the newborn (PPHN) is a disorder of unsuccessful circulatory transition at birth [1]. It is characterized by the extrapulmonary shunting of blood at the level of the patent ductus arteriosus (PDA) and patent foramen ovale (PFO), resulting in hypoxemia. Labile hypoxemia and a preductal to postductal oxygen saturation by the pulse oximetry (SpO_2_) gradient are classic features of PPHN. The pre- and postductal gradient is thought to be secondary to right-to-left or bidirectional shunting across the PDA [2]. This gradient can be measured using simultaneous arterial blood gases (ABG) or by pulse oximetry.

As part of the screening for critical congenital heart disease (CCHD), an oxygen saturation by pulse oximetry (SpO_2_) gradient of > 3% [3] is taken as an indication for further testing [4]. This screening method often detects conditions such as PPHN in addition to CCHD [5,6,7]. However, there are no studies directly measuring the relation of ductal shunts to simultaneous pre- and postductal blood gases.

We evaluated the relationship between the direction of ductal shunt and blood gas parameters in simultaneous preductal (right carotid) and postductal (umbilical artery) samples in a lamb model of meconium aspiration syndrome (MAS) with PPHN. We hypothesized that the presence of a bidirectional or right-to-left shunt at the PDA will be associated with a clinically significant gradient between the pre- and postductal partial arterial oxygen tension (PaO_2_) and saturation of arterial oxygen (SaO_2_) concentrations.

## 2. Materials and Methods

The study protocol was approved by the Institutional Animal Care and Use Committee (IACUC, protocol #20267) at the University of California Davis (UCD) and has been described in detail previously [8,9,10]. This institution is accredited by the Association for Assessment and Accreditation of Laboratory Animal Care, International (AAALAC). UCD has an Animal Welfare Assurance on file with the Office of Laboratory Animal Welfare (OLAW). The Assurance Number is D16-00272 (A3433-01). The IACUC is constituted in accordance with the U.S. Public Health Service (PHS) Animal Welfare Policy and includes a member of the public and a non-scientist.

### 2.1. Animal Preparation

Time-dated near-term (138–141 day gestation; term ~145 days) pregnant ewes were bred by Van Laningham Farm, Arbuckle, CA, USA. Following an overnight fast, the ewe was sedated with intravenous propofol or diazepam and ketamine. The ewe was then intubated with a 9.5-mm cuffed endotracheal tube (ETT), provided general anesthesia with 2–4% inhaled isoflurane, and continuously monitored with a pulse oximeter and an end-tidal carbon dioxide (ETCO_2_) monitor. Following a laparotomy, the fetal lamb was partially exteriorized and intubated with a 4.5-mm cuffed endotracheal tube (ETT). The fetal lung fluid in the ETT was passively drained by lowering the head and, thereafter, the ETT was occluded to prevent gas exchange during gasping, following asphyxiation by cord occlusion. Under maternal anesthesia, and after infiltrating the site with subcutaneous bupivacaine, an incision was made to place a catheter in the lamb’s right carotid artery for the measurement of blood pressures and the collection of blood samples. The right jugular vein was catheterized for fluid and medication administration. A 3-mm flow probe (Transonic, Ithaca, NY, USA) was placed around the left carotid to measure the blood flow. A left thoracotomy was performed for placement of flow probes to measure blood flow in the left pulmonary artery (Q_P_; 4-mm probe) and the ductus arteriosus (Q_DA_; 6-mm probe). Finally, both the thoracotomy and neck incisions were surgically closed. The baseline hemodynamic measurements and arterial blood gases were recorded.

### 2.2. Experimental Protocol

After instrumentation and baseline measurements, a 30 mL syringe was attached to the ETT and a 20% solution of meconium in amniotic fluid (approximately 5 mL/kg) was instilled into the ETT. Intravenous analgesic support was started prior to cord clamping. Acute prenatal asphyxiation was induced by occluding the umbilical cord for five minutes or until the heart rate decreased below 40 beats per minute. During this time period, the meconium solution was “spontaneously” aspirated during gasping and distributed into the lungs. The umbilical cord compression was relieved for two minutes to allow for hemodynamic recovery, followed by a second five-minute cord occlusion interval. Following meconium aspiration, the umbilical cord was tied and cut, and the lambs were delivered.

The lambs were transferred to a radiant warmer and mechanically ventilated. The peak inspiratory pressure (PIP) was adjusted based on the exhaled tidal volume, ETCO_2_, and partial arterial carbon dioxide (PaCO_2_). A catheter was then placed in the umbilical artery to collect postductal blood samples. A pulse oximeter was placed on the right forelimb for continuous saturation monitoring (SpO_2_). A second pulse oximeter was placed around a hind limb for postductal SpO_2_ measurements. The inspired oxygen concentration was adjusted to achieve a preductal SpO_2_ as per the Neonatal Resuscitation Program guidelines for the first 15 min. Subsequent management was based on the consensus statement from the European Pediatric Pulmonary Vascular Disease Network (EPPVDN) [11]. We targeted a preductal SpO_2_ between 91% and 95%, a PaO_2_ between 50 and 70 mmHg, and a PaCO_2_ between 45 and 60 mmHg. Lambs were monitored for up to six hours, whereupon the blood gases and hemodynamic data were analyzed at 15 min intervals. At the completion of the study period, the lambs were euthanized.

### 2.3. Statistical Analysis

Blood gases were analyzed using a blood gas analyzer (Radiometer ABL90 FLEX, Denmark), and hemodynamic variables were continuously recorded using computer acquisition and analysis software (BIOPAC Systems, Goleta, CA, USA). The ductal flow (systolic maximum, diastolic minimum, and mean) was recorded simultaneously at the time of the pre- and postductal blood gas sampling. Blood gas variables, specifically SaO_2_, and flows are expressed as means with standard deviations (SDs). By convention, the right-to-left ductal flow was labeled negative and the left-to-right flow was labeled positive on the acquisition device. The ductal flow was recorded as the minimum (diastolic flow), mean, and maximum (systolic flow). Throughout the analysis, the right-to-left or bidirectional ductal shunting was defined by values with negative (right-to-left) flow either during the diastole, systole, or both phases of the cardiac cycle. The left-to-right ductal shunting was defined by positive flow throughout the cardiac cycle. We also evaluated the hemodynamic and gas exchange implications of a pre- and postductal SaO_2_ gradient of ≥ 3% and < 3%. The pre- and postductal comparisons were analyzed by a two-tailed, paired Student’s t-test. Comparisons between the right-to-left/bidirectional and left-to-right ductal shunt groups were analyzed using a two-tailed, Student’s t-test with unequal variances. Statistical significance was defined as *p* < 0.01.

## 3. Results

A total of 129 hemodynamic and blood gas time-points were compiled and sorted into right-to-left/bidirectional and left-to-right ductal shunt groups based on the ductal blood flow direction (Table 1).

The blood gas and hemodynamic parameters were also classified based on the pre- to postductal SaO_2_ gradient (Table 2). The hemoglobin, preductal SpO_2_, pH, blood lactate, fractional inspired oxygen (FiO_2_), and mean airway pressures were not significantly different between these two groups.

### 3.1. Shunting and Ductal Blood Flow

**Figure 1:**
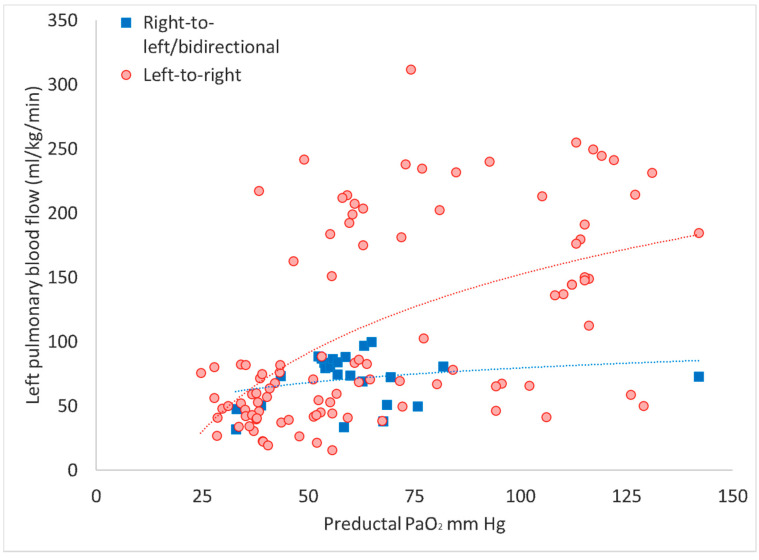
Left pulmonary blood flow (Q_P_) plotted against the preductal partial arterial oxygen tension (PaO_2_). Left Q_P_ is significantly lower when there is bidirectional shunt through the ductus arteriosus compared to when the shunting is from left to right (aorta towards pulmonary artery).

Blood gas samples drawn during the presence of right-to-left (or bidirectional) ductal shunting (as defined in the methodology) had significantly lower postductal PaO_2_ values and significantly higher preductal and postductal PaCO_2_ values compared to the samples drawn in the presence of left-to-right shunting (Table 1). Only two instances of exclusive right-to-left shunting throughout the cardiac cycle were observed in the right-to-left (or bidirectional) ductal shunting, and hence this group is henceforth referred as the bidirectional shunt group. The left-to-right ductal flow is an important contributor to pulmonary blood flow, and samples with a bidirectional shunt had a lower Q_P_ when plotted against the preductal PaO_2_ (Figure 1).

Mean systemic blood pressure was significantly lower in the bidirectional group compared to the left-to-right ductal shunt group (53 ± 12 vs. 68 ± 10 mm Hg, *p* < 0.01). The left Q_CA_ was significantly lower in the bidirectional shunt group (12.1 ± 3.9 vs. 14.6 ± 5.7 mL/kg/min, *p* < 0.01).

**Figure 2:**
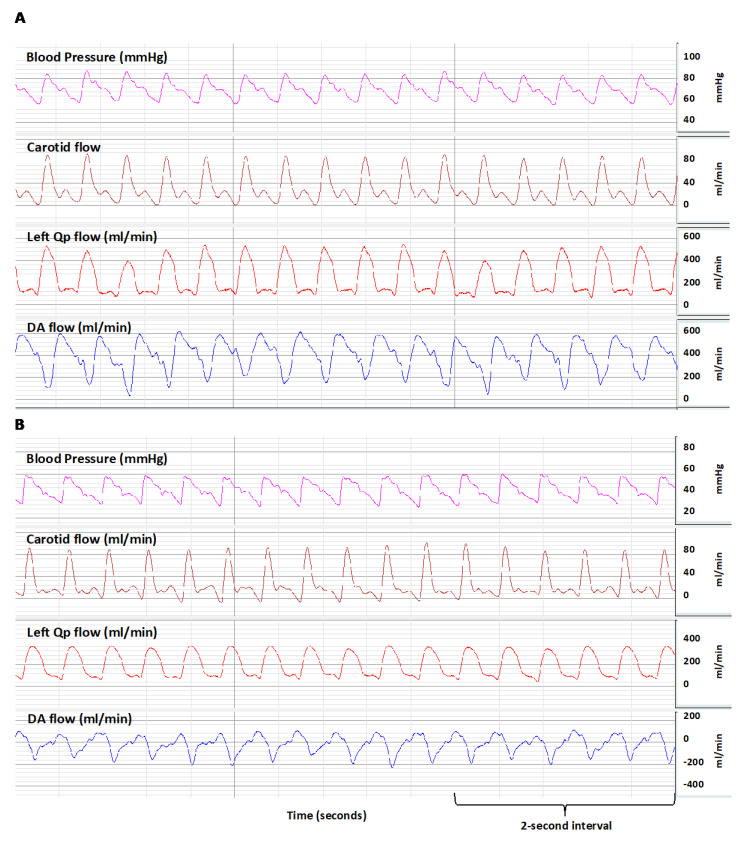
Screenshot of the left pulmonary (Q_P_) blood flow, left carotid blood flow (Q_CA_), and ductal arteriosus (DA) blood flow showing a transition from left-to-right shunting (top, (**A**)) and bidirectional shunting (bottom, (**B**)) through the DA. The left Q_P_ is lower when the shunt is bidirectional.

Analysis within groups revealed that the postductal arterial oxygen content (CaO_2_) and SaO_2_ were significantly lower than their preductal counterparts during bidirectional shunting (Table 1). There was no significant difference between the pre- and postductal CaO_2_ and SaO_2_ values in the presence of left-to-right shunting (Table 1). When evaluating for the oxygenation index (OI), the preductal samples were significantly lower than the postductal samples regardless of the group/shunting directionality, with no difference in OI between the bidirectional group compared to the left-to-right ductal shunt group (Table 1). Oxygen delivery to the brain was calculated by multiplying the carotid blood flow with the preductal arterial oxygen content (Q_CA_ × CaO_2_/100). Oxygen consumption by the brain was calculated using the following equation Q_CA_ × (CaO_2_ − CvO_2_)/100, where CaO_2_ is the carotid arterial oxygen content and CvO_2_ is the jugular venous oxygen content. The brain oxygen delivery and oxygen consumption by the brain were lower in the bidirectional shunt group (Table 1). A representative BIOPAC image of bidirectional shunt and left-to-right shunt are shown in Figure 2.

### 3.2. Shunting and Pre- and Postductal Difference in SaO_2_

Samples with a pre- and postductal oxygen saturation gradient of ≥ 3% had a lower pre- and postductal SaO_2_, lower preductal PaO_2_, and higher PaCO_2_ compared to the < 3% group (Table 2). The mean airway pressure and FiO_2_ were similar between the two groups. When analyzing the pre- and postductal arterial saturation differences, 56% of samples with a bidirectional or right-to-left shunt had a pre- and postductal saturation difference of < 3% (Table 2). However, only 11% of the samples containing a ≥ 3% difference were associated with exclusive left-to-right shunts (Table 2).

**Figure 3:**
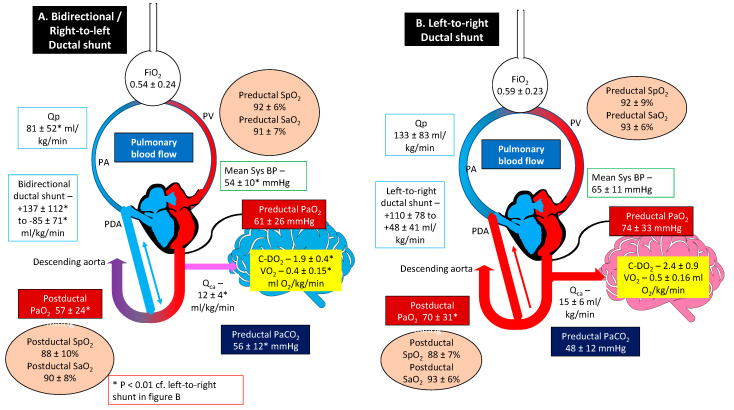
Graphical abstract illustrating hemodynamic and oxygen saturation differences comparing the bidirectional to left-to-right ductus arteriosus shunting in a lamb model of meconium aspiration syndrome and pulmonary hypertension. Lambs that experience a bidirectional ductal shunt have a significantly lower left pulmonary arterial blood flow (Q_P_), left carotid blood flow (Q_CA_), mean systolic blood pressure (Sys BP), cerebral oxygen delivery (C-DO_2_), and oxygen consumption (VO_2_) compared to left-to-right ductal shunting. FiO_2_ = fraction of inspired oxygen; PaO_2_/PaCO_2_ = partial arterial oxygen/carbon dioxide tension; SaO_2_ = saturation of arterial oxygen. Copyright Satyan Lakshminrusimha.

A graphic summary of the results is presented in Figure 3.

## 4. Discussion

Extrapulmonary shunt at the PDA and PFO level leading to hypoxemia is characteristic of PPHN. Birth asphyxia with meconium aspiration is a common cause of PPHN in term infants [12]. The presence of right-to-left ductal shunting is inferred by the presence of an oxygenation gradient between the pre- and postductal regions. In this study, using a model of birth asphyxia, meconium aspiration syndrome, and secondary PPHN, we demonstrate that bidirectional ductal shunting with hypoxemia can occur without a significant pre- to postductal oxygenation gradient.

There are several limitations to this study. The degree of hypoxemic respiratory failure was mild to moderate. We did not evaluate shunting at the PFO level. Significant right-to-left shunting at the PFO may potentially reduce the pre- and postductal oxygen gradient. We did not consistently measure the postductal SpO_2_ in all lambs due to the lack of an adequate number of pulse oximeters during multiple, simultaneous studies. The degree of right-to-left shunting may potentially be more significant in the presence of severe hypoxemia and PPHN. The ovine model of severe PPHN can be induced by antenatal ductal ligation [13]. However, it is not possible to assess ductal shunting in this model. We did not measure the pulmonary arterial pressure in these studies and did not assess the severity of PPHN. We did document hypoxemia, low pulmonary blood flow, and bidirectional or right-to-left shunting (2 samples) in this study, and these findings are suggestive of PPHN. Finally, we did not statistically correct for multiple values obtained from the same lamb. There was widespread fluctuation in the direction of shunting, pulmonary blood flow, and blood gases in the same lamb with time.

The novel aspect of the study is the simultaneous measurement of ductal, pulmonary, and carotid flows along with pre- and postductal blood gases. A right-to-left or bidirectional flow at the ductus is associated with a significant reduction in the pulmonary blood flow, leading to significantly lower PaO_2_/FiO_2_ ratios in these lambs. The presence of a bidirectional shunt was also associated with lower systemic blood pressures and a higher PaCO_2_. Despite high PaCO_2_ concentrations, the carotid blood flow, oxygen delivery, and oxygen consumption by the brain were significantly lower in the presence of a bidirectional shunt. This suggests that oxygen delivery to the brain is compromised in the presence of bidirectional shunt in this model.

In neonates with PPHN the absence of a pre- and postductal oxygenation gradient is thought to be due to the closure of the PDA or an exclusively left-to-right shunt at the PDA [1]. The current study shows that even in the presence of a low pulmonary blood flow and bidirectional ductal shunt, there may not be a significant saturation or PaO_2_ gradient between the preductal and postductal regions. The oxygen saturation in the lower limb is dependent on the admixture of pulmonary arterial blood (with a mixed venous oxygen saturation of 70% ± 9% in our study) and preductal arterial blood (with SaO_2_ of 90.9% ± 6.5%). Using the shunt equation, the right-to-left effective ductal shunt only contributes to 6.6% of the descending aortic flow in lambs with a bidirectional ductal flow. The presence of a saturation difference between the preductal and postductal samples is a reliable indicator of PPHN and a bidirectional or right-to-left shunt (89% of instances—Table 2). However, the absence of a saturation gradient between the preductal and postductal samples does not rule out PPHN despite the presence of hypoxemia with compromised oxygen delivery to the brain.

## 5. Conclusions

In lambs with parenchymal lung disease and secondary PPHN, the presence of a pre- and postductal oxygenation gradient was indicative of a right-to-left or bidirectional shunt. However, the lack of a pre- and postductal oxygenation gradient does not rule out bidirectional shunt. In clinical situations with parenchymal lung disease, the clinical absence of a pre- and postductal oxygenation gradient should not be considered to suggest the absence of PPHN, and higher emphasis should be placed on obtaining echocardiography to confirm the diagnosis.

## Figures and Tables

**Table 1:** Blood gas parameters comparing lambs with a right-to-left or bidirectional ductal shunt vs. exclusively left-to-right ductal shunt.

Shunting	Right-to-Left or Bidirectional Ductal Shunting	Left-to-Right Ductal Shunting
	(*n* = 80)	(*n* = 49)
SpO_2_ (%), preductal	92 ± 6	92 ± 9
SaO_2_ (%), preductal	91 ± 7 ^◊^	93 ± 6
SaO_2_ (%), postductal	90 ± 8	93 ± 6
SaO_2_ (%), postductal **	88 ± 10	88 ± 7
PaO_2_ (mmHg), preductal	61 ± 26 ^◊^	74 ± 33 ^◊^
PaO_2_ (mmHg), postductal	57 ± 24 *	70 ± 31
PaCO_2_ (mmHg), preductal	56 ± 12 *	48 ± 12 ^◊^
PaCO_2_ (mmHg), postductal	56 ± 12 *	50 ± 12
Mean Q_CA_ (mL/kg/min)	12 ± 4 *	15 ± 6
Mean systemic blood pressure (mmHg)	54 ± 10 *	65 ± 11
Mean Q_DA_ (mL/kg/min)	32 ± 7 *	84 ± 57
Max Q_DA_ (mL/kg/min)	137 ± 112	110 ± 78
Min Q_DA_ (mL/kg/min)	−85 ± 71 *	48 ± 41
Mean Left Q_P_ (mL/kg/min)	81 ± 52 *	133 ± 83
CaO_2_ (mLO_2_/dL), preductal	16 ± 2	16 ± 3
CaO_2_ (mLO_2_/dL), postductal	16 ± 2	16 ± 3
Lactate (mmol/L)	3.2 ± 1.2	3.7 ± 1.5
pH	7.2 ± 0.1	7.2 ± 0.1
Hb (g/dL)	13.1 ± 1.2	12.8 ± 1.8
FiO_2_	0.54 ± 0.24	0.59 ± 0.23
Mean Airway Pressure (cm H_2_O)	10 ± 1.8	10 ± 1.5
Oxygenation Index (OI), preductal	11 ± 2.5	10 ± 6.1
Oxygenation Index (OI), postductal	11 ± 2.9	10 ± 6.2
PaO_2_/FiO_2_ ratio, preductal	126 ± 60 ^◊^	137 ± 72 ^◊^
PaO_2_/FiO_2_ ratio, postductal	119 ± 32	132 ± 69
A-a DO_2_ (preductal)	269 ± 162	286 ± 179
A-a DO_2_ (postductal)	273 ± 163	287 ± 179
Brain O_2_ delivery (mL/kg/min)	1.9 ± 0.4 *	2.4 ± 0.9
Brain oxygen consumption (mL/kg/min)	0.4 ± 0.15 *	0.5 ± 0.16

Data shown as mean ± SD. A-a DO_2_ = Alveolar-arterial oxygen gradient; CaO_2_ = content of arterial oxygen; FiO_2_ = fraction of inspired oxygen; PaCO_2_ = partial tension of arterial carbon dioxide; PaO_2_ = partial tension of arterial oxygen; Q_CA_ = carotid blood flow; Q_DA_ = ductal blood flow; Q_P_ = pulmonary arterial blood flow; SpO_2_ = saturation of pulsed oxyhemoglobin; SaO_2_ = saturation of arterial oxygenation * *p* < 0.01 cf. left-to-right group and ^◊^ *p* < 0.01 cf. postductal. ** Postductal SpO_2_ values were available for only 13 samples in the bidirectional shunt group and 8 samples in the left-to-right group.

**Table 2:** Blood gas parameters comparing greater than or equal to 3% differences and less than 3% differences between the pre- and postductal oxygen saturations.

Oxygen Saturation Gradient	ΔSaO_2_ ≥ 3%	ΔSaO_2_ < 3%
	(*n* = 18)	(*n* = 111)
SaO_2_ (%), preductal	86 ± 10 *^,◊^	93 ± 5 ^◊^
SaO_2_ (%), postductal	81 ± 10	92 ± 5
PaO_2_ (mmHg), preductal	51 ± 17 *^,◊^	68 ± 30 ^◊^
PaO_2_ (mmHg), postductal	42 ± 12	65 ± 28
FiO_2_	0.61 ± 0.25	0.56 ± 0.24
Mean Airway Pressure (cm H_2_O)	11 ± 2.3	10 ± 1.6
Oxygenation Index (OI), preductal	14 ± 6.0 ^◊^	10 ± 5.4 ^◊^
Oxygenation Index (OI), postductal	16 ± 6.4 *	10 ± 5.6
PaCO_2_ (mmHg), preductal	59 ± 10 *	52 ± 12
PaCO_2_ (mmHg), postductal	62 ± 9.2 *	52 ± 12.3
Mean Q_CA_ (mL/kg/min)	10 ± 2.5 *	13 ± 5.0
Mean Q_DA_ (mL/kg/min)	60 ± 69	50 ± 55
Max Q_DA_ (mL/kg/min)	233 ± 95 *	109 ± 91
Min Q_DA_ (mL/kg/min)	−96 ± 99 *	−25 ± 84
Mean Left Q_P_ (mL/kg/min)	71 ± 66	106 ± 70
Samples with bidirectional ductal or right-to-left shunt	16 (89%)	62 (56%)

Data shown as mean ± SD. Q_CA_ = carotid blood flow; Q_DA_ = ductal blood flow; Q_P_ = pulmonary arterial blood flow. * *p* < 0.01 cf. left-to-right group (ΔSaO_2_ < 3%) and ^◊^ *p* < 0.01 cf. postductal.
